# Vaginal Flatus and the Associated Risk Factors in Iranian Women: A Main Research Article

**DOI:** 10.5402/2012/802648

**Published:** 2012-05-20

**Authors:** Firoozeh Veisi, Negin Rezavand, Maryam Zangeneh, Shohreh Malekkhosravi, Mansour Rezaei

**Affiliations:** ^1^The High Risk Pregnancy Research Center, Imam Reza Hospital, Kermanshah University of Medical Sciences, Kermanshah 6714415333, Iran; ^2^Obstetrics and Gynecology Department, Kermanshah University of Medical Sciences, Kermanshah 6714415333, Iran; ^3^Department of Biostatistics, Kermanshah University of Medical Sciences, Kermanshah 6714415333, Iran

## Abstract

*Objective*. The aim of the study was to determine the prevalence of vaginal flatus and some related risk factors in Iranian women. *Methods*. After conducting a pilot study, a sample size of 1000 subjects of 18 to 80 years was determined; of those 58 were unable to cooperate for various reasons. Age, parity, marital status, birth history, body mass index, and the weight of the largest newborn were collected. After a full gynecologic examination looking for pelvic organ prolapse, patients were asked about vaginal flatus and the frequency and time of occurrence. The results were studied using the chi-square test and independent *t*-test considering an alpha error of less than 0.05. *Results*. The prevalence of vaginal flatus was mainly 20% in this study, but embarrassment was observed in 5.7% of these women. 4% in the group were with no history of sexual contact (virgin). Vaginal flatus mostly started after vaginal delivery (45%) or spontaneously (34%); however, it was also reported after cesarean section and other pelvic operations. The most common activity leading to vaginal flatus was intercourse (54%); however, the time which had resulted in more inconvenience for the patients was during physical activities (92%). BMI and age were significantly lower in the patients (*P* < 0.0001). The grade of prolapse was lower in the patients (*P* < 0.0001). *Conclusion*. Low age, low body mass index, and vaginal delivery can affect the incidence of a noisy vagina.

## 1. Introduction

There is evidence of women's genital prolapse and its treatment in the remained works of the Egyptians around 1550 BC. Hippocrates around 400 BC would hang the patient from her leg for the treatment of prolapse and moved her up and down like a pendulum to reduce the size of prolapse [1]. 

Pelvic floor disorders include urinary incontinence, pelvic organ prolapse, incontinence, and abnormal sensory and discharge incontinence in the urinary and intestinal tracts. A regional study in USA has shown that about 10% of women would experience surgery in their lives for urinary incontinence, prolapse, or both, and the number of surgeries is two or more in approximately 30% [2].

 Pelvic floor disorders are common and are related to gender, race, age, pregnancy, vaginal delivery, instrumental delivery, episiotomy, infant head circumference, obesity, constipation, or connective tissue disorders [3–6]. Vaginal delivery is considered as a risk factor for pelvic prolapses, however, has not been confirmed in all the studies; the studies have mentioned that the cesarean section does not prevent urinary or fecal incontinence [7, 8]. It has been shown that the lowest risk of prolapse is in Black women and Hispanic women are at the greatest risk [9]. It is also reported that the rate of high-grade prolapse was higher in the Asian women [10]. In some studies, the prevalence of pelvic floor disorders varies in different races, but has similar risk factors [10, 11]. In various studies conducted in Iran, the incidence of pelvic prolapses has been reported to be between 50% and 53% [12].

Vaginal flatus is one of the other pelvic disorders that have uncertian causes, which has received less attention (perhaps due to not being uncomfortable or life threatening). Vaginal Flatus has been described as an uncomfortable situation with a negative impact on the quality of life of women of all ages, which not only creates social and psychological problems, but also causes impairment in religious duty practice. There have been few studies in this area and each may use a different term to describe it including vaginal wind, vaginal flatus, vaginal noise, or noisy vagina [13–17].

Krissi et al. have considered the prevalence of this complaint uncertain in their study, which might be due to the small sample size. In addition they have considered vaginal delivery as the most important risk factor for its occurrence. They had also noted that more research needs to be done to study the situation and assess the risk factors, prevalence, and the treatment [13]. Hsu introduced a patient with a history of two times cesarean section and no apparent prolapse who suffered this distressing situation and concluded that vaginal delivery is not the only cause of this problem, and cesarean section can also be involved [18]. Jeffrey and colleagues reported that their patient was suffering from vaginal gas passage without any obvious pelvic prolapse; by using cubic Pessary, the complaint improved [19]. However, in the developed countries, the patients have less reported this problem for various reasons; therefore, there are limited studies in this area; hence, Krissi and colleagues recruited only six women in their study. The vaginal flatus is embarrassing to Iranian women, because it leads to their isolation from public and it is in contrast to their religious customs. As a result, we decided to study the prevalence of this complaint and some associated risk factors.

## 2. Methods

This study has received an ethical approval from the research council of Kermanshah University of Medical Science to be conducted at the Imam Reza Hospital.

It was decided to carry out a cross-sectional study of women who were referred which were 1000 patients and the study was carried out between April and June 2009. Participants signed informed consent and completed the research questionnairs at the visit time.

Initially a pilot study over two weeks identified 11 cases of vaginal flatus amongst 80 referred patients. Hence, a sample size of 1000 patient was calculated to ascertain the associated risk factors. Of the 1000 participants, 58 were declined for individual reasons. Inclusion criteria, were women 18 to 80 years, married, single, virgin, nulriparous, and multiparous. The women with these characters and referred to our clinic were eligible for the study. After compelete explanation to participants and getting informed consent, they were enrolled in the study.

 Exclusion criteria including the inability to answer questions (58 patients), pregnancy, less than six months since the last delivery, patient's inability to identify the original location of gas passage (anal or vaginal), gynecological cancers, and existence of rectal and vaginal fistula. Demographic data such as age, parity, and heaviest baby born weight were recorded.

Their weight and height were also recorded to determine body mass index. All patients underwent gynecologic examination to identify a possible Pelvic Organ Prolapse Quantification. The POP-Q examination was performed by a study gynecologist and her assistant at a university hospital in lithotomy position and valsalva maneuver. It was explained to participants the differences between anal and vaginal gas passage. Then they were asked about any complaint of vaginal gas passage and if confirmed, they were questioned about its frequency.

Patients with this complaint were divided into two groups: the group experiencing the situation only a few times a week and the group experiencing it several times a day. Patients were also questioned about the time of occurrence including during daily activities such as getting up or sitting, praying, exercising, and intercourse. Meanwhile, the patients were asked to remember the event that triggered the problem (vaginal delivery, cesarean section, hysterectomy, or pelvic floor reconstruction or spontaneously).

Finally, the independent sample *t*-test and chi-square were used for quantitative and qualitative variables, respectively. An alpha error less than 0.05 was considered significant.

## 3. Results

In total 1000 women were eligible and 58 were excluded. 942 women were examined. Vaginal flatus was reported in 188 (20%) of participants. Of these women reporting vaginal flatus, 101 (54%) were in intercourse and most of them considered it normal and had no complaint. The embarrassing vaginal flatus was observed in 54 (5.7%) of participants (Table 1).

The mean age of women with the complaint was almost 34 years, which was less than those without the complaint (*P* < 0.0001). The BMI of the affected group was lower (24.61 versus 25.84) (*P* < 0.0001). Parity was not different among the women with or without the complaint. The grade of pelvic prolapse was surprisingly lower in the women with the problem. Prolapse grade ≥3 was 12% in affected versus 36% in nonaffected women (*P* < 0.0001). 8 of the cases were unmarried women who had never experienced sexual contacts (for conventionally and religious reasons virgin girls have no intercourse). The prevalence of the complaint was 71% in women with a history of vaginal delivery, while it was 16%  in those with a history of cesarean section, and 4% in patients with a history of both. Most cases were seen in women of reproductive age and the large infant delivery was partly effective in the incidence of the complaint (*P* = 0.032) (Table 1).

The most important event leading factor to vaginal flatus was normal vaginal delivery (45%), although, many were having the problem spontaneously (37.6%). However, there were people who were affected after caesarean section, hysterectomy or pelvic floor repair. This shows that vaginal flatus is a multifactorial problem (Table 2). Although the patients were having the problem most commonly after intercourse (54%), 92% of the participants who got vaginal flatus during the activity were embarrassed about this situation. It happened one or two times a week in some patients up to several times a day in other sever cases (Table 3).

## 4. Discussion

The issue of vaginal flatus does appear to be underresearched; however, gynecologists need to deal with it though it is less discussed in the medical literature. Vaginal flatus is an involuntary gas passage through the genital tract. This may cause social difficulties or interfere with the marital relationship. These patients appear less in the public due to fear of embarrassment and are sometimes blamed by their husbands and have difficulties in their religious practice (prayer).

Nokes and colleagues reported vaginal air in 11% of healthy women [14] but Hadar et al. observed intravaginal air in the pelvic CT scan of women to be associated with pelvic malignancies and other pelvic pathology [15]. Small amounts of air seen on only one section without distention of the vagina were common [14]. The passage of gas from vaginal origin does not cause a problem in most cases, but some people are annoyed and uncomfortable. In this study, the prevalence of vaginal flatus was 20%, which is perhaps too much, but should be considered in most women; vaginal flatus was done during intercourse and only 5.7% is to refer them for treatment.

 In the present study, 20% of cases occurred during the daily activities which can cause inconvenience for the individual. The current study is unlike the study by Slieker-Ten Hove et al. who reported the prevalence of vaginal wind to be 12.8% and the number of bothered patients to be very low. The difference between our study and Sliekers can be due to cultural reasons. 

 She considered vaginal wind as a vaginal pelvic floor complaint with the underlying mechanisms to be unknown. But 72% of women declared that this situation has created a little distress for them [16]. In the study by Krissi, due to limited number of patients (250), the incidence of vaginal flatus was uncertain [13]. In the study by Krissi et al., the mean age of women complaining of vaginal flatus was 32.8 years; in our study, it was a little more (about 34 years) but less than the control group.

Slieker-Ten Hove et al. reports the mean age of women as 56.5 years, which can be related to the higher age range in their study population (45–85 years) [16].

 The mean body mass index (BMI) in the patients with vaginal flatus was significantly less at 24.6 in our study compared to 25.8 in the group without complaints (*P* value < 0.0001). The six patients of Krissi et al. also had a low mean BMI of 24 [13]. It is possible that due to less body mass index, the vaginal walls are not supported adequately to prevent them from folding over each other. There are several reports showing that parity has a direct association with prolapse; the higher the parity, the more likely the pelvic organ prolapse [20]. In our study, no association was observed between the parity and the incidence of vaginal flatus (*P* = 0.368).

Although in the current study vaginal flatus was far more common in the patients with intercourse, the presence of the complaint in the virgin girls (4%) was interesting, because the virgin girls has no sexual relationship and delivery.

 The difference between married and virgin women might be due to the virgins less presenting to gynecologic clinics than married women. In addition, Slieker-Ten Hove et al. did not find any association between sexual intercourse and vaginal flatus [16]. It appears that factors other than those of pregnancy, childbirth, sex, and surgery are involved in the occurrence of vaginal flatus.

The girls who have not labor and delivery, do not have pelvic muscle weakness either. About vaginal flatus in girls, the overactivity of the pelvic muscle can be mentioned. Uncoordinated pelvic floor muscle activities may lead to increased vaginal flatus in virgins. Our results found no significant differences between cesarean and normal vaginal delivery in prevalence of vaginal flatus (*P* > 0.0001). Vaginal birth is a direct cause of prolapsed [21] but no vaginal flatus.

 However, some of the patients in the control group claimed a history of previous vaginal flatus which was resolved after the second delivery or posterior colporrhaphy (the data not shown here). Surprisingly, vaginal flatus was seen in the single girls and women with a history of childbirth through cesarean section, as well. And even some patients reported the onset of the symptoms to be after hysterectomy or pelvic floor surgeries; this issue makes the recognition of the risk factors and, as a result, the treatment difficult. Slieker-Ten Hove used pelvic organ prolapse scoring system (POP-Q) and studied 9 locations in the pelvis; he concluded that the anatomic changes in the Bp point in the posterior wall of vagina from -3 to the whole length of vagina may be the main source of the disorder. He considered high-grade prolapse, high-parity and solid feces incontinence to be the most important associated risk factors of the disease. On the contrary in our study vaginal flatus was seen in women with low-grade prolapse (grades 1 and 2 were 65%) than high grade (*P* > 0.0001). The weight of the neonate affected the incidence of vaginal flatus, with 100 grams difference (*P* = 0.032). It is said that the length of perinea is shorter in the Asian race women compared with the Europeans. Theoretically, this can result in lack of proper vaginal closure.

It should be mentioned that there were women who had experienced the problem in the past, but had improved following a vaginal delivery or pelvic floor repair (these were considered in the disorder-free group). According to the present study, the risk factors of noisy vagina (Garrulitas vulvae) included being slim (resulting in low pelvic muscle strength), lower age, history of vaginal delivery, and giving birth to a large neonate.

## 5. Conclusion

Our results show that the prevalence of vaginal flatus is 20% and the related risk factors are low age, low BMI and low prolapse. Our study was based on clinical examinations; we propose pelvic MRI for the future studies in order to evaluate the pelvic floor functions and disorders. This will lead to more valuable and more accurate results. We have not measured the length of the vagina and perinea in our cohort.

## Figures and Tables

**Table 1:** Distribution of demographic data in the study group.

	With vaginal flatus	Without vaginal flatus	*P* value
Total Number	188 (20%)	754 (80%)	—
With embarrassment:	54 (5.7%)		
Age	34 ± 10.8	37 ± 12.4	<0.0001
Parity	2.5 ± 2.15	2.6 ± 2.14	0.368
No prolapse	43 (23%)	147 (19.5%)	
Pelvic prolapse grade = 1	84/188 (45%)	164/754 (22%)	
Pelvic prolapse grade = 2	38/188 (20%)	171/754 (23%)	<0.0001
Pelvic prolapse grade ≥ 3	23/188 (12%)	272/754 (36%)	
BMI (kg/m^2^)	24.6 ± 4.4	25.9 ± 3.7	
Marital status			
Virgin	8 (4%)	68 (12% )	>0.0001
Married	168 (89%)	592 (78.5%)	
Divorced or widow	12 (6%)	94 (12% )	
Kind of prior delivery			
Vaginal delivery	134 (71%)	392 (52% )	>0.0001
Cesarean section	30 (16%)	123 (16%)	
Both of them	8 (4%)	42 (6%)	
Others: virgin, abortion, nulipar	16 (8.5%)	227 (30% )	
Menopause	37 (20% )	118 (16%)	—
Largest baby (kg)	3.3 ± 0.6	3.2 ± 0.6	0.032

**Table 2:** Distribution of vaginal flatus after each event.

Normal vaginal delivery	84 (45%)
Cesarean section	23 (12%)
Hysterectomy	9 (5%)
Prior pelvic repair	8 (4%)
Spontaneously	64 (34%)

**Table 3:** Frequency distribution of the activities associated with vaginal flatus.

	Vaginal noise	Bothering situation
Intercourse	101/188 (54%)	10/101 (10%)
Daily activities	71/188 (38%)	65/71 (92%)
Exercise	15/188 (8%)	8/15 (53%)

## References

[1] Rock J, Wilbur H (2008). *Te Linde’s Operative Gynecology*.

[2] Olsen AL, Smith VJ, Bergstrom JO, Colling JC, Clark AL (1997). Epidemiology of surgically managed pelvic organ prolapse and urinary incontinence. *Obstetrics and Gynecology*.

[3] MacLennan AH, Taylor AW, Wilson DH, Wilson D (2000). The prevalence of pelvic floor disorders and their relationship to gender, age, parity and mode of delivery. *British Journal of Obstetrics and Gynaecology*.

[4] Gurel H, Gurel SA (1999). Pelvic relaxation and associated risk factors: the results of regression analysis. *Acta Obstetricia et Gynecologica Scandinavica*.

[5] Yang X, Zhang HX, Yu HY, Gao XL, Yang HX, Dong Y (2010). The prevalence of fecal incontinence in primiparous postpartum Chinese women. *European Journal of Obstetrics & Gynecology and Reproductive Biology*.

[6] Miedel A, Tegerstedt G, Maehie-Schmidt M, Nyren O, Hammarstrom M (2009). Nonobstetric risk factors for symptomatic pelvic organ prolapse. *Obstetrics & Gynecology*.

[7] Borello-France D, Burgio KL, Richter HE (2006). Fecal and urinary incontinence in primiparous women. *Obstetrics and Gynecology*.

[8] Lal M, Mann CH, Callender R, Radley S (2003). Does cesarean delivery prevent anal incontinence?. *Obstetrics and Gynecology*.

[9] Hendrix SL, Clark A, Nygaard I, Aragaki A, Barnabei V, McTiernan A (2002). Pelvic organ prolapse in the Women’s Health Initiative: gravity and gravidity. *American Journal of Obstetrics and Gynecology*.

[10] Sewell CA, Chang E, Sultana CJ (2007). Prevalence of genital prolapse in 3 ethnic groups. *Journal of Reproductive Medicine for the Obstetrician and Gynecologist*.

[11] Kim S, Harvey MA, Johnston S (2005). A review of the epidemiology and pathophysiology of pelvic floor dysfunction: do racial differences matter?. *Journal of Obstetrics and Gynaecology Canada*.

[12] Garshasbi A, Faghih-Zadeh S, Falah N (2006). The status of pelvic supporting organs in a population of Iranian women 18–68 years of age and possible related factors. *Archives of Iranian Medicine*.

[13] Krissi H, Medina C, Stanton SL (2003). Vaginal wind—a new pelvic symptom. *International Urogynecology Journal and Pelvic Floor Dysfunction*.

[14] Nokes SR, Martinez CR, Arrington JA, Dauito R (1986). Significance of vaginal air on computed tomography. *Journal of Computer Assisted Tomography*.

[15] Hadar H, Kornreich L, Heifets M, Herskovits P, Horev G (1999). Air in vagina. Indicator of intrapelvic pathology on CT. *Acta Radiologica*.

[16] Slieker-Ten Hove MC, Pool-Goudzwaard AL, Eijkemans MJC, Steegers-Theunissen RPM, Burger CW, Vierhout ME (2009). Vaginal noise: prevalence, bother and risk factors in a general female population aged 45–85 years. *International Urogynecology Journal and Pelvic Floor Dysfunction*.

[17] Attapattu JAF (1995). Garrulitas vulvae: a report of six cases. *Journal of Reproductive Medicine for the Obstetrician and Gynecologist*.

[18] Hsu S (2007). Vaginal wind—a treatment option. *International Urogynecology Journal and Pelvic Floor Dysfunction*.

[19] Jeffery S, Franco A, Fynes M (2008). Vaginal wind—the cube pessary as a solution?. *International Urogynecology Journal and Pelvic Floor Dysfunction*.

[20] Nygaard I, Barber MD, Burgio KL (2008). Prevalence of symptomatic pelvic floor disorders in US women. *Journal of the American Medical Association*.

[21] Larsson C, Kallen K, Andolf E (2009). Cesarean section and risk of pelvic organ prolapse: a nested case control study. *American Journal of Obstetrics & Gynecology*.

